# Mind–body-medicine in oncology—from patient needs to tailored programs and interventions: a cross-sectional study

**DOI:** 10.3389/fpsyg.2023.1140693

**Published:** 2023-07-06

**Authors:** Jonas Leonhardt, Marcela Winkler, Anne Kollikowski, Lisa Schiffmann, Anne Quenzer, Hermann Einsele, Claudia Löffler

**Affiliations:** ^1^Department of Internal Medicine II, University Hospital of Wuerzburg, Wuerzburg, Germany; ^2^Department of Natural and Integrative Medicine, Robert-Bosch-Hospital, Stuttgart, Germany; ^3^Comprehensive Cancer Center, University Hospital of Wuerzburg, Wuerzburg, Germany; ^4^Department of Gynecology and Obstetrics, University Hospital of Wuerzburg, Wuerzburg, Germany

**Keywords:** lifestyle habits, symptom burden, individual mind state, motivational level, stress

## Abstract

**Introduction:**

National and international guidelines recommend early integration of evidence-based multimodal interventions and programs, especially with a focus on relaxation techniques and other Mind–Body-based methods to maintain the quality of life of oncology patients, improve treatment tolerability, and promote healthy lifestyle behaviors. Consequently, we aim to understand what drives patients and how they navigate integrative medicine to best advise them. This study aimed to detect possible topics of particular interest to patients and identify the patient groups that could benefit most from further programs. Furthermore, we aimed to investigate if patients are open-minded toward integrative oncology concepts and learn about their motivational level to maintain or change behavior.

**Methods:**

Between August 2019 and October 2020 we surveyed patients undergoing oncological therapy in a university oncological outpatient center using a custom-developed questionnaire based on established Mind–Body Medicine concepts.

**Results:**

We included 294 patients with various cancers. More than half reported problems sleeping through (61%) and 42% felt stressed frequently, invariably rating this as detrimental to their health. Moreover, a slight majority (52%) felt physically limited due to their disease and only 30% performed defined exercise programs. Women were significantly more likely to feel stressed and reported with alarming frequency that they often feel “everything was up to them.” The 40–65-year-olds reported significantly less restful sleep, more stress and were more dissatisfied with their situation. However, this group already used natural remedies most frequently and was most often motivated to use relaxation techniques in the next 6 months. The lower the perceived individual energy level (EL), the less frequently patients did sport, the more frequently they felt their disease impaired their activity, mostly feeling stressed and tense. We also found significant associations between negative emotions/thoughts and the variables “sleep,” “use of relaxation techniques,” “personal stress perception,” and “successful lifestyle modification.”

**Conclusion:**

Mind–Body programs that focus on patient’s individual resources, with tools to explore impairing patterns of self-perception and cognitive biases, can be a valuable resource for oncology patients and should therefore be part of an integrative medical treatment concept.

## 1. Introduction

Numerous studies prove the sustained interest of Europeans and especially Germans in complementary and alternative medicine (CAM) (Kemppainen et al., 2018; Fjær et al., 2020; Lederer et al., 2021). Patients desire to strengthen personal resources, play an active role in coping with their disease, receive holistic care, and improve the side effects of their therapy (Davidson et al., 2002; Boon et al., 2003; Verhoef et al., 2005; Evans et al., 2007; Huebner et al., 2014; Klafke et al., 2014). In contrast to alternative methods, integrative oncology as a future model of evidence-based complementary medicine stands for a meaningful addition to conventional therapies (Deng et al., 2013; Witt et al., 2017). National and international guidelines recommend early integration of evidence-based multimodal interventions and programs to maintain the quality of life of oncology patients, improve treatment tolerability, and promote healthy lifestyle behaviors (Greenlee et al., 2017; Jeitler et al., 2017; Voiß et al., 2020; Deutsche Krebsgesellschaft, 2021). A focus within these multimodal concepts is on Mind–Body-Medicine (MBM) interventions and techniques. The National Institutes of Health (NIH) describes MBM as a discipline that focuses on the nature of the interactions that link the brain, the body, the mind, and behavior to one another and how emotional, mental, social, spiritual, experiential, and behavioral factors can directly influence health (Esch and Stefano, 2022).

Therefore, we wanted to determine subjectively unmet needs for integrative counseling and treatment services among cancer patients at the Comprehensive Cancer Center of the University Hospital of Wuerzburg. Moreover, we aimed to investigate if patients are open-minded toward integrative oncology concepts and to learn how motivated they are to change behavior. We also wanted to better understand those topics of particular interest to patients and identify those who could benefit most from further programs. We hypothesized that individual mindset at the time of the survey might both influence how stressed and burdened patients feel and affect their motivation to change behavior. Hence, we focused specifically on individual emotional/mental state, as well as inquired about interest in Mind–Body interventions, which we judged to be an appropriate evidence-based resource.

## 2. Materials and methods

### 2.1. Participants and recruitment

Patients suffering from a hematological or solid tumor and undergoing outpatient treatment were recruited by an MD student and physicians at the Interdisciplinary Outpatient Departments for Chemotherapy for Internal Medicine and Gynecology of Wuerzburg University Hospital. The patients had to be at least 18 years old. Otherwise, there were no exclusion criteria. After detailed explanation of the questionnaire by an MD student, each patient signed the consent form, and written informed consent regarding data protection. Consent could be withdrawn at any time during and after the survey.

### 2.2. Data collection

A MD student extracted general patient information (e.g., stage of the disease, medication, clinical course of therapy) from medical records. The number of different medication prescribed daily at the time of the survey was taken into account, whereby the oncological therapy itself was not counted. Medication prescribed more than once a day were only taken into account once in the evaluation. On-demand medication, as well as medication that should be taken less frequently than once a day, were not included in the evaluation.

### 2.3. Questionnaire

We used a custom-developed questionnaire based on a Mind–Body-Medicine Day Care Clinic program, first published in 2013 and intended to support patients cope with their disease and establish a healthy lifestyle (Paul et al., 2013; Dobos et al., 2015). The Day Care Clinic program combines elements of the MBM Cancer Program of the Benson-Henry Mind/Body Medical Institute at Harvard Medical School, techniques from the Mindfulness-Based Stress Reduction (MBSR) program, as well as defined methods of self-regulation and self-care (Kabat-Zinn, 1982; Kabat-Zinn, 1990; Benson, 1999). It also focusses on the patient’s individual resources, based on the salutogenic model to “guide health promotion” (Mittelmark et al., 2017). Accordingly, relaxation techniques, exercise, cognitive restructuring, nutrition, and social support are important elements.

Patients were asked to provide feedback on the following (* indicates dichotomous items): a.o. sleep*; daily rhythms; perceived energy level on a scale from 0 (lowest imaginable energy level) to 10 (highest imaginable energy-level); use of breathing* and/or relaxation techniques and personal stress perception*; dietary habits; sports activity; use of natural remedies; social support*; thoughts, feelings, and attitudes. In order to better understand the relationship between thoughts, feelings, attitudes and wellbeing, as well as the motivation to develop healthy habits, patients were asked to assess which statements they could identify with from five statements suggesting either a rather optimistic or a rather pessimistic emotional alignment. Regarding health-promoting behavior, we asked how long this could already be maintained and whether and when patients could imagine changing their behavior. The data was collected paper based. Supplementary Table S2 provides detailed information on the set of questions, as well as on missing data.

### 2.4. Statement of ethics

Patients’ written, informed consent could be withdrawn at any time during and after the survey. The study was approved by the Ethics Committee of the Julius-Maximilian-University Wuerzburg on 15 April 2019 (study number 12/19-me) and performed according to the Declaration of Helsinki.

### 2.5. Statistical analysis

The results were statistically analyzed using the data processing program IBM SPSS Statistics version 25 for Windows (IBM SPSS Statistics for Windows, 2017). We aimed to include at least 250 patients. Given the explorative nature and novelty of the study, we performed no sample size calculation. However, since the aim of the study was to find out which parameters might be relevant for patients, we decided to use this broad approach in order to estimate effect sizes of associated parameters for future studies.

Besides descriptive statistics analyses for absolute and relative frequencies, observed numbers and means, we determined normal distributions using the Kolmogorov–Smirnov test and visual inspection of histograms. A subgroup analysis compared participants who provided positive statements to those who provided negative statements. Further subgroup analyses compared gender (male, female), and age categories (18–39 years, 40–65 years, and over 65 years old) (see Tables 1–3). The classification of the age categories was based on the model of Sender and Zabokrtsky, especially concerning younger patients (Sender and Zabokrtsky, 2015). To examine if a low energy level might interfere with initiating and maintaining lifestyle modifications, we divided patients into the following groups according to their perceived energy level (EL): low EL (0–3/10), medium EL (4–6/10), and high EL (7–10/10). In all calculations chi-square test was used to detect association and differences. The significance level was set at *p* < 0.05 for all tests (Bühl, 2019). Effect size was expressed by Phi coefficent (φ) and Cramers V to measure association between two variables. Subgroup analysis calculations primarily used 2 × 2 crosstabs for gender, 2 × 3 crosstabs for age, and 2 × 2 crosstabs for positive and negative statements. In few calculations larger crosstabs were used.

**Table 1:** Clinical characteristics of patients, oncological data, physical/mental wellbeing.

		♀ 148	♂ 146	18–39 years	40–65 years	>65 years	*n* = 294
*Patient characteristic and oncological data*							
Age	18–39 years	6 (67%)	3 (33%)				9 (3%)
	40–65 years	80 (56%)	62 (44%)				142 (48%)
	>65 years	62 (43%)	81 (57%)				143 (49%)
Intention to treat	Palliative care	99 (48%)	108 (52%)	5 (2%)	92 (44%)	110 (53%)	207 (70%)
	Curative care	38 (44%)	49 (56%)	4 (5%)	50 (58%)	33 (38%)	87 (30%)
Medication	≥5 agents per day	63 (44%)	80 (56%)	4 (3%)	56 (39%)	83 (58%)	143 (49%)
	<5 agents per day	85 (56%)	66 (44%)	5 (3%)	86 (57%)	60 (40%)	151 (51%)
*Breathing*							
“I am aware that my breathing is related to my inner tension.”		103 (51%)	99 (49%)	5 (2.5%)	102 (51%)	95 (47%)	202 (72%)
“I consciously pay attention to my breathing at least once a day.”		77 (51%)	73 (49%)	4 (3%)	70 (47%)	76 (51%)	150 (54%)
“If not, “I plan to start in the next 30 days.”		33 (38%)	20 (62%)	2 (4%)	29 (55%)	22 (42%)	53 (28%)
*Physical and mental wellbeing*							
Sleep and daily rhythms	Sleep classified as restful	93 (51%)	90 (49%)	6 (3%)	73 (40%)	104 (57%)	183 (68%)
	Sleep-onset insomnia	48 (61%)	31 (39%)	2 (3%)	42 (53%)	35 (44%)	79 (29%)
	Sleep maintenance insomnia	77 (47%)	88 (53%)	3 (2%)	82 (50%)	80 (49%)	165 (61%)
	Night rest period, mean (h), (mean all patients: 8.8 h)	8.8	8.7	8.7	8.5	9	224 (76%)
Perceived energy level (0–10)	Mean (0–10) (mean all patients: 5.7)	5.9	5.4	6.4	5.5	5.9	267 (90%)
Personal stress perception	Often feels tense, burdened, and stressed	71 (61%)	46 (39%)	5 (4%)	68 (58%)	44 (38%)	117 (42%)
	Has the impression that stress has a negative impact on health	87 (56%)	68 (44%)	6 (4%)	84 (54%)	65 (42%)	155 (56%)
	Is convinced that they can influence inner tension	117 (50%)	116 (50%)	7 (3%)	123 (53%)	103 (44%)	233 (84%)

When adding up all percentages of the columns, there are deviations to 100% due to rounding.

**Table 2:** Patients’ lifestyle habits: presentation of results comparatively between men and women, and comparatively between different age groups.

		♀ 148	♂ 146	18–39 years	40–65 years	> 65 years	*n* = 294
*Lifestyle habits*							
Use of relaxation techniques*	For more than 6 months	19 (54%)	16 (46%)	0	16 (46%)	19 (54%)	35 (13%)
	For less than 6 months	11 (65%)	6 (35%)	1 (6%)	9 (53%)	7 (41%)	17 (6%)
	Plans to start	29 (62%)	18 (38%)	3 (6%)	34 (72%)	10 (21%)	47 (17%)
	Would not like to start	46 (36%)	81 (64%)	4 (3%)	54 (43%)	69 (54%)	127 (46%)
	On demand	33 (34%)	17 (66%)	1 (2%)	25 (50%)	24 (48%)	50 (18%)
Healthy diet**	For more than 6 months	107 (54%)	92 (46%)	7 (4%)	97 (49%)	95 (48%)	199 (72%)
	For less than 6 months	17 (50%)	17 (50%)	0	23 (68%)	11 (32%)	34 (12%)
	Plans to start	15 (63%)	38 (47%)	1 (4%)	11 (46%)	12 (50%)	24 (9%)
	Would not like to start	4 (21%)	15 (79%)	1 (5%)	7 (37%)	11 (58%)	19 (7%)
Exercise and sport***	For more than 6 months	92 (47%)	102 (53%)	9 (5%)	95 (49%)	90 (46%)	194 (73%)
	For less than 6 months	12 (71%)	5 (29%)	0	11 (65%)	6 (35%)	17 (6%)
	Plans to start	23 (59%)	16 (41%)	0	21 (54%)	18 (46%)	39 (14%)
	Would not like to start	8 (40%)	12 (60%)	0	8 (40%)	12 (60%)	20 (7%)
Use of natural remedies****	For more than 6 months	29 (45%)	36 (55%)	0	35 (54%)	30 (46%)	65 (24%)
	For less than 6 months	10 (71%)	4 (29%)	0	10 (71%)	4 (29%)	14 (5%)
	Plans to start	39 (61%)	25 (39%)	7 (11%)	34 (53%)	23 (36%)	64 (23%)
	Would not like to start	60 (46%)	70 (54%)	2 (2%)	58 (45%)	70 (54%)	130 (48%)

*“Do you regularly perform conscious relaxation exercises such as autogenic training, progressive muscle relaxation, visualization or meditation?”

**“Do you eat a mostly healthy diet (e.g., low in meat and saturated fat with lots of fruits and vegetables, good sources of protein, whole grains, and good fats)?”

***“Do you currently exercise regularly, i.e., for 30 min, at least 4 days per week (walking, hiking, cycling…)?”

****“Do you regularly use natural remedies such as water treatments, herbal medicines, sauna sessions or even massages?”

When adding up all percentages of the data in rows, there could arise deviations to 100% due to rounding.

**Table 3:** Patients’ lifestyle habits: behavior change already implemented or intention to change behavior within each subgroup.

		♀ 148	♂ 146	18–39 years (*n* = 9)	40–65 years (*n* = 142)	> 65 years (*n* = 143)
*Lifestyle habits*						
Use of relaxation techniques*	Patients who have answered the question	138	138	9	138	129
	For more than 6 months	19 (14%)	16 (12%)	0	16 (12%)	19 (15%)
	For less than 6 months	11 (8%)	6 (4%)	1 (11%)	9 (7%)	7 (5%)
	Plans to start	29 (21%)	18 (13%)	3 (33%)	34 (25%)	10 (8%)
	Would not like to start	46 (33%)	81 (59%)	4 (44%)	54 (39%)	69 (53%)
	On demand	33 (24%)	17 (12%)	1 (11%)	25 (18%)	24 (19%)
Healthy diet**	Patients who have answered the question	143	133	9	138	129
	For more than 6 months	107 (75%)	92 (69%)	7 (78%)	97 (70%)	95 (74%)
	For less than 6 months	17 (12%)	17 (13%)	0	23 (17%)	11 (9%)
	Plans to start	15 (10%)	9 (7%)	1 (11%)	11 (8%)	12 (10%)
	Would not like to start	4 (3%)	15 (11%)	1 (11%)	7 (5%)	11 (9%)
Exercise and sport***	Patients who have answered the question	135	135	9	135	126
	For more than 6 months	92 (68%)	102 (76%)	9 (100%)	95 (70%)	90 (71%)
	For less than 6 months	12 (9%)	5 (4%)	0	11 (8%)	6 (5%)
	Plans to start	23 (17%)	16 (11%)	0	21 (16%)	18 (15%)
	Would not like to start	8 (6%)	12 (9%)	0	8 (6%)	12 (10%)
Use of natural remedies****	Patients who have answered the question	138	135	9	137	127
	For more than 6 months	29 (21%)	36 (27%)	0	35 (26%)	30 (24%)
	For less than 6 months	10 (7%)	4 (3%)	0	10 (7%)	4 (3%)
	Plans to start	39 (28%)	25 (19%)	7 (78%)	34 (25%)	23 (18%)
	Would not like to start	60 (43%)	70 (52%)	2 (22%)	58 (42%)	70 (55%)

*“Do you regularly perform conscious relaxation exercises such as autogenic training, progressive muscle relaxation, visualization or meditation?”

**“Do you eat a mostly healthy diet (e.g., low in meat and saturated fat with lots of fruits and vegetables, good sources of protein, whole grains, and good fats)?”

***“Do you currently exercise regularly, i.e., for 30 min, at least 4 days per week (walking, hiking, cycling...)?”

****“Do you regularly use natural remedies such as water treatments, herbal medicines, sauna sessions or even massages?”

When adding up all percentages of the columns, there could arise deviations to 100% due to rounding.

## 3. Results

### 3.1. Patient characteristics

A total of 294 patients with approximately equal numbers of women and men were surveyed. The mean age was 63.9 ± 11.9 years. Slightly less than half had hematological, one third gastrointestinal and less than one-fifth had gynecological malignancies (see Supplementary Table S1). Seventy percent (*n* = 207) were in palliative and 30% (*n* = 87) in curative therapy with a prospect of cure (Table 1).

### 3.2. Physical and mental wellbeing and lifestyle habits

Although more than half reported sleep-through disturbances (*n* = 165; 61%), the majority rated sleep quality as good and restful (*n* = 183; 68%). However, the perceived average daytime energy level was rated at 5.7 out of 10. Forty-two percent reported feeling tense and stressed frequently, with more than half rating this detrimental to their health. Although the majority reported being conscious of a connection between breathing and inner tension, only half of the patients paid attention to their breathing at least once a day and about one third were motivated to start within the next 30 days (Table 1). The vast majority felt well integrated into their social network of friends and family (*n* = 276, 96%), were confident they could rely on caregivers in challenging situations (*n* = 274, 98%) and enjoyed work/daily tasks (*n* = 244, 87%).

Two thirds (*n* = 187, 66%) reported they already had successful experiences with lifestyle changes in the past, although more than one third reported they had often quickly fallen back into bad habits (*n* = 95, 37%). Tables 1–3 illustrate reported mental and physical wellbeing, as well as health behaviors, and summarize the motivation for behavior change regarding various lifestyle habits.

Concerning physical activity, over half reported feeling physically limited due to their disease (*n* = 146, 52%). Although a total of 78% (*n* = 211) stated that they were physically active at least 4 days a week for 30 min each, patients notably reported particularly about increased everyday activities (*n* = 248, 88%) and only 30% (*n* = 85) stated in an additional question that they performed defined exercise programs. Furthermore, a relevant proportion equated relaxing activities, such as sleeping or reading, with structured relaxation techniques.

### 3.3. Health-promoting behavior by patient characteristics—thoughts, feelings, and attitudes

**Figure 1:**
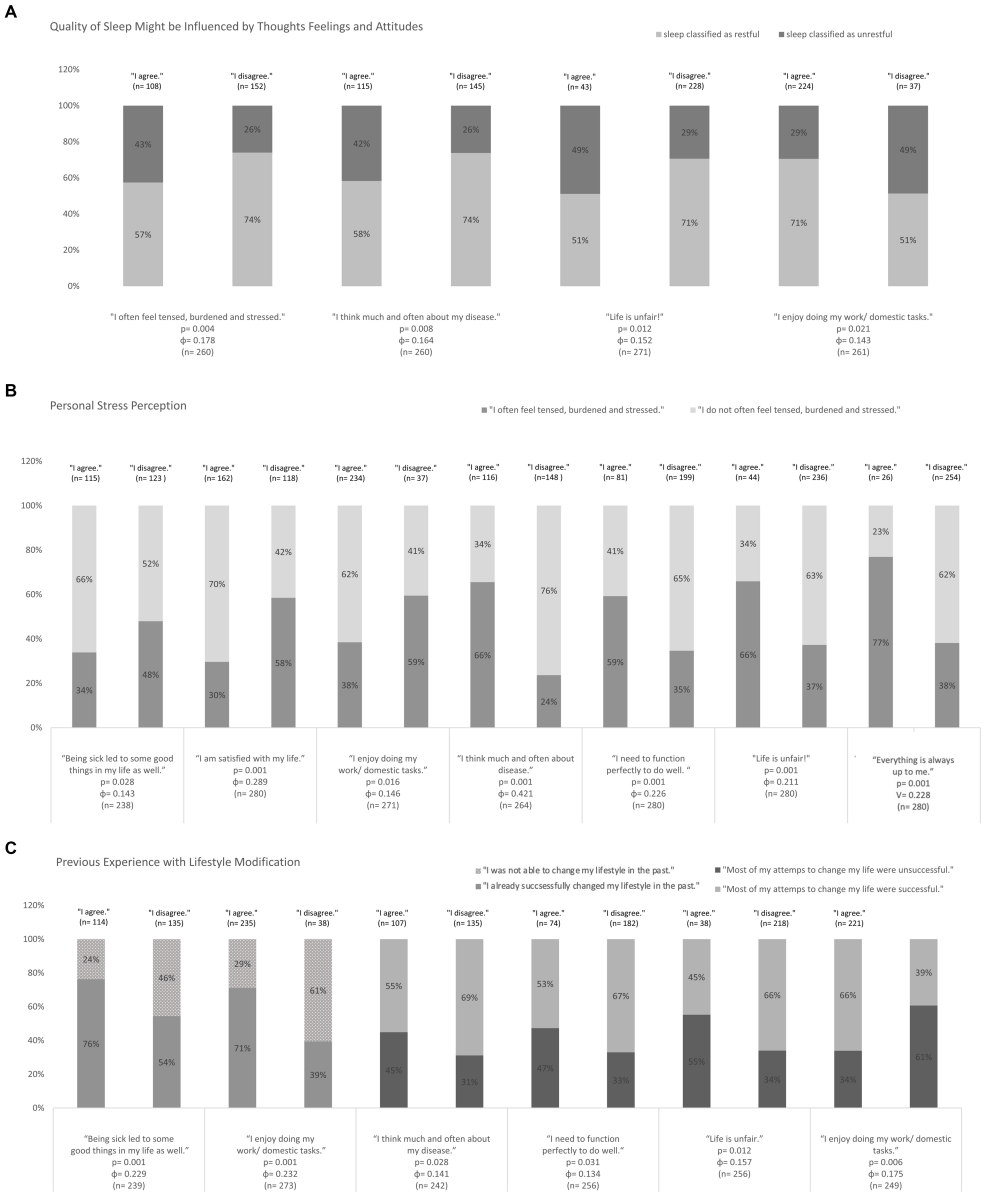
Influence of individual mindset on the variables of **(A)** sleep, **(B)** stress perception, and **(C)** experience with lifestyle modifications. Chi-square test were used, *p*-values and Phi-coefficient is given in the Diagramm (n indicates the number of patients, who have answered the question, respectively).

Depending on the current emotional/mental mind state, we analyzed differences regarding “sleep,” “personal stress perception,” and “successful lifestyle modification” (Figure 1). Chi-square test with 2 × 2 crosstabs was used. Patients who reported restful sleep were significantly less likely to feel tensed and stressed, less likely to think about their disease, less likely to feel that “life is unfair,” and more likely to enjoy their daily tasks. In addition, patients who felt less stressed were significantly more likely to identify with statements that indicate a more optimistic emotional alignment. In addition, an optimistic attitude was associated with significantly more positive experiences in terms of sustainable lifestyle changes.

### 3.4. Health-promoting behavior by patient characteristics—gender and age

**Figure 2:**
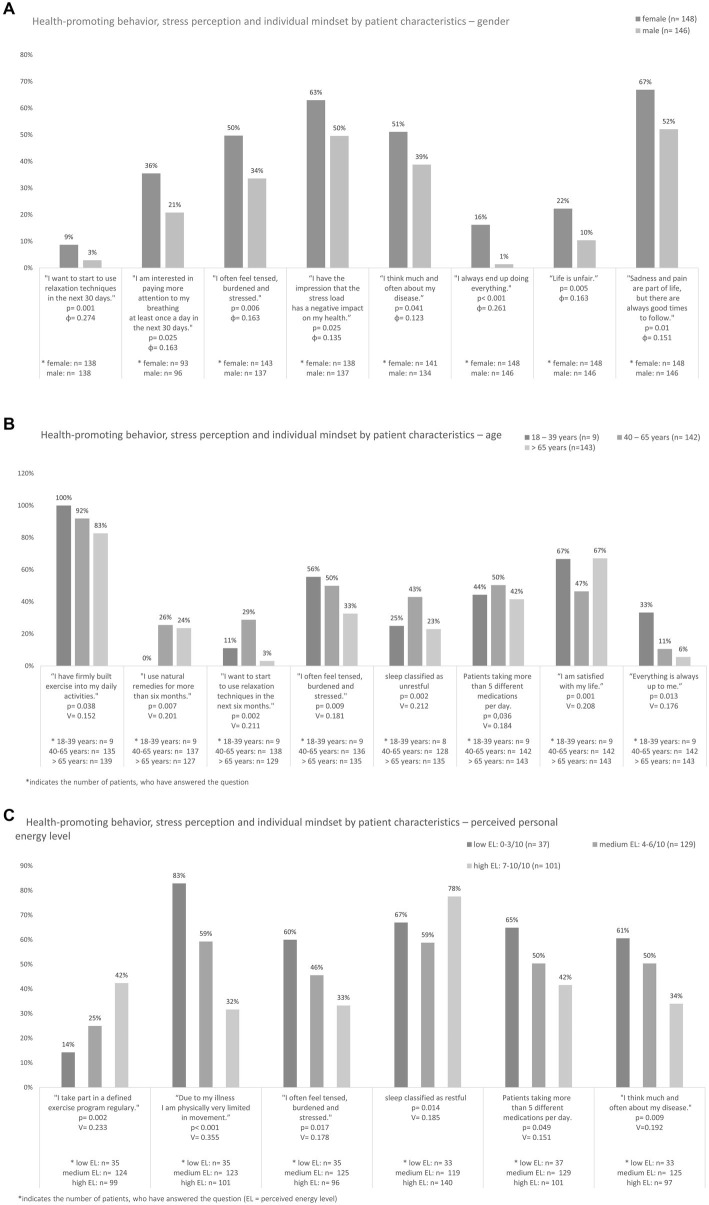
Health-promoting behavior by patient characteristics of **(A)** gender, **(B)** age, and **(C)** perceived personal energy level. Chi-square test was used, *p*-values and Cramers V or Phi-coefficient given in the Diagramm.

Significant findings were also achieved when looking at gender and age (Tables 1–3; Figure 2) using Chi-square tests. To analyze gender differences, we primarily used 2 × 2 crosstabs, regarding age we primarily used 2 × 3 crosstabs. We found a significant association between gender and use of relaxation techniques [*x*^2^(5) = 20.8, *p* < 0.001, *V* = 0.274]. Women were significantly more likely to feel stressed, more often concerned about their wellbeing regarding most lifestyle habits and more motivated to change behavior. They were significantly more interested in paying more attention to their breathing at least once a day in the next 30 days [*x*^2^(1) = 5.0, *p* = 0.025, ɸ = 0.163]. Furthermore, women were already twice as likely to use relaxation techniques and even three times as likely to use a relaxation technique in the next 30 days. However, significantly more women picked negative statements such as to “*I think much and often about my disease*” [1.4 times as often, *x*^2^(1) = 4.2, *p* = 0.041, ɸ = 0.123], “*I always end up doing everything*” [12 times as often, *x*^2^(1) = 20.1, *p* < 0.001, ɸ = 0.261] and *“life is unfair”* [twice as often, *x*^2^(1) = 7.8, *p* = 0.005, ɸ = 0,163]. More women than men could identify with the statement “*sadness and pain are part of life, but there are always good times to follow*” [1.3 times, *x*^2^(1) = 6.6, *p* = 0.01, ɸ = 0.151]. Regarding the variable age, we found significant differences concerning “sleep” [*x*^2^(2) = 12.2, *p* = 0.002, *V* = 0.212], “use of relaxation techniques” [*x*^2^(10) = 24.6, *p* = 0.002, *V* = 0.211], “exercise into daily activities” [*x*^2^(2) = 6.7, *p* = 0.038, *V* = 0.152] and “use of natural remedies” [*x*^2^(8) = 22.1, *p* = 0.007, *V* = 0.285]. The 40-65-year-olds reported significantly less restful sleep, more stress and were more dissatisfied with their own situation compared to the other age groups. This group already used natural remedies most frequently and was most often motivated to use relaxation techniques in the next 6 months. The older the patients, the less frequently they reported regular physical activity. The younger the patients, the more frequently they stated, “*I always end up doing everything*.”

### 3.5. Health-promoting behavior by patient characteristics—perceived personal energy level

We found significant associations between the perceived personal energy level and the variables “sleep,” “exercise,” “personal stress perception” and “feeling, thoughts and attitudes” (Figure 2). Chi-square test and 2 × 3 crosstabs were primarily used. The higher the EL, the more often patients reported restful sleep [*x*^2^(2) = 8.6, *p* = 0.014, *V* = 0.185]. Moreover, patients with high EL were two times less likely to think about their disease [*x*^2^(2) = 9.4, *p* = 0.009, *V* = 0.192] and two times more likely to report being satisfied with their situation [*x*^2^(2) = 11.4, *p* = 0.003, *V* = 0.207]. The lower the EL, the less frequently patients were active in sports [*x*^2^(2) = 13.8, *p* = 0.002, *V* = 0.223], the more frequently they felt their disease impaired their activity [*x*^2^(2) = 32.7, *p* < 0.001, *V* = 0.355], and also felt stressed and tense most of the time [*x*^2^(2) = 8.1, *p* = 0.017, *V* = 0.178]. This patient group with the lower EL was significantly more likely to rely on more than 5 medications per day [*x*^2^(2) = 6.0, *p* = 0.049, *V* = 0.151].

## 4. Discussion

### 4.1. Physical and mental wellbeing and lifestyle habits: we should use the full potential of relaxation techniques such as breathing exercises

Two out of 5 patients reported feeling stressed regularly. This matches results of other studies that demonstrated an average up to 52% of patients feel limited by increased disease-associated stress (Zabora et al., 2001; Mehnert et al., 2018).

In the first months after a cancer diagnosis, studies have shown that both chronic and episodic stress are independent predictors of the increase in distressing physical symptoms such as pain and fatigue (Harris et al., 2017). Additionally, stress levels appear to be the strongest predictor of quality of life 2 years after diagnosis (Härtl et al., 2010). It is all the more striking that almost half of the patients do not yet use any relaxation techniques and reported no current interest in starting promptly, although 56% reported stress could have a negative effect on their health.

Oppegaard et al. (2021) could demonstrate impressively that anxiety profiles are associated with stress, resilience and symptom severity in outpatients receiving chemotherapy. A recent study examining the effects of a group-based integrative oncology program to build resilience and improve quality of life in cancer patients revealed that patients with anxiety and low initial resilience benefited most (Savaş et al., 2022). Regarding relaxation methods, breathing exercises play an important role as they are easy to learn and available ubiquitously. There is convincing evidence that concentrative breathing exercises contribute to improved stress regulation and can reduce anxiety (Jerath et al., 2015; Soni and Muniyandi, 2019). A majority (74%) was aware of the connection between breathing and inner tension, with only one third reporting they would like to focus more on their own breathing in the future. Especially informal quick exercise sessions, such as mindful awareness of breathing together with easy-to-follow explanations about the connections between breathing, stress, and physical wellbeing, should be in an integrative treatment program. Moreover, patients need to know that relaxing activities such as sleeping or even reading are not the same as structured relaxation techniques. Not to forget, it is important to encourage patients to discuss the use relaxation techniques/mind–body-based interventions with their care providers. Patients might not give information about use of mind–body medicine out of fear of physician criticism (Davis et al., 2012). On the other hand, the attitude of the physician could also have an influence on the attitude of the patients. There exists first evidence, that physician’s lifestyle might influence the prescription of healthy habits to breast cancer patients (Cangussú et al., 2022).

In addition, these results show that there is a considerable need not only for multimodal group services, but also for intensive psycho-oncological support in individual settings.

### 4.2. Patients need to perform more defined exercise programs

Our results showed that 52% of the respondents felt physically limited, despite regular physical activity which is recommended as being at least 30 min of moderate to intense aerobic activity three times a week and 20–30 min of resistance training twice a week (Campbell et al., 2019). Any kind of physical activity in everyday life also including defined exercise programs is important, especially to achieve the most important goal: avoiding physical inactivity (Campbell et al., 2019). But it seems that the duration and intensity of physical activity should have been higher for these patients to reduce physical limitation, especially since only 30% reported doing defined physical activity programs. Our results also showed patients with a low EL less likely to be physically active and more likely to feel impaired, stressed, and tense. In contrast, patients with a higher EL reported sleeping better, were more satisfied, and thought less about their illness. Therefore, patients should be directed to practice defined supervised physical activity programs to address specific needs and physical problems (Schmitz et al., 2019; Depenbusch et al., 2020). Oncologists play a significant role as the interface with the patient and should therefore regularly assess the patients’ current physical activity, advise them on their current and desired physical activity and communicate the importance of regular exercise. If this is not possible due to time constraints, they should at least refer patients to appropriate physical activity programs and/or exercise therapy professionals (Schmitz et al., 2019). A recent study has shown that physical exercise counseling in cancer patients has an impact on their physical activity and, depending on previous activity levels, directly related to higher physical activity after diagnosis (Depenbusch et al., 2020). In addition, compelling evidence exists for supervised exercise therapy compared to unsupervised interventions (Buffart et al., 2017). In conclusion, the least physicians can do is follow the so-called “call-to-action” (Schmitz et al., 2019).

### 4.3. Health-promoting behavior by patient characteristics—thoughts, feelings, and attitudes might influence health-promoting behavior

More than two-thirds of patients had reported positive experiences with lifestyle modifications, but 37% indicated they had often quickly fallen back into bad habits.

Behavior change is easier to maintain if achievable goals are chosen and the person is confident they have good resources to achieve them. Life experiences and personal core values can influence how people react in difficult situations and particularly how patients come to terms with their disease (Savioni and Triberti, 2020; Sevild et al., 2020). We therefore hypothesized that individual mindset at the time of the survey may have influenced how stressed and burdened patients felt and their motivation to change their behavior (Bandura, 1982; Ashford et al., 2010). Patients indicating a more optimistic emotional alignment were significantly more likely to report successful and sustainable lifestyle modifications compared to patients with a presumably more pessimistic mindset. Moreover, patients who felt pleasure in their daily tasks and reported thinking less often about their disease were significantly less likely to feel life was unfair and to experience sleep disturbances (Roberts and Duong, 2014; Wang et al., 2015; Scott et al., 2021). Although we found an association between patients’ satisfaction with their own situation and their perceived stress level, this patient group was also significantly more likely to report already regularly practicing relaxation techniques including perceptions and patterns of evaluation. Mindfulness, for example, means non-judgmental awareness of the present moment. Studies have demonstrated the effect of regular relaxation and mindfulness practice on stress, satisfaction and gratitude (Hoffman et al., 2012; Cramer et al., 2013; Love et al., 2019; Norelli et al., 2022). Additionally, there is convincing evidence that mindfulness-based interventions and especially integrative day-care programs focusing on MBM may benefit patients with anxiety and depressiveness (Witt et al., 2015; Haller et al., 2017; Jin et al., 2019; Li et al., 2019; Parmentier et al., 2019). Since approximately one third of cancer patients suffers from depressiveness, Mind–Body procedures should be encouraged early, especially for patients who are more likely to identify with pessimistic thoughts (Krebber et al., 2014). In addition, it also makes sense to use the resource of psycho-oncological counseling even more, e.g., focusing on cognitive biases and behavioral therapy approaches.

### 4.4. Gender-specific differences require adapted support

As observed in other studies, women were significantly more motivated to change their lifestyle habits (Verhoef et al., 2005; Voiß et al., 2019). However, another important finding was that especially women felt stressed significantly more often and were also concerned that this could have a negative impact on their health. Other studies provide similar results (Bergerot et al., 2015; Fischer et al., 2022). Women were also able to identify significantly more often with rather pessimistic statements and reported with alarming frequency that they often feel “everything was up to them.” Other studies also show that, e.g., breast cancer patients feel insufficiently supported in their social networks and that younger female patients particularly suffered more frequently from depression and anxiety (Linden et al., 2012). We therefore hypothesize that this patient group could particularly benefit from interventions oriented toward behavioral therapy, supporting the cultivation of a positive mindset, but also from socio-medical counseling (Salakari et al., 2017; Sousa Rodrigues Guedes et al., 2020; Zainal et al., 2021). Evidence for both single interventions and multimodal programs continues to grow (Latte-Naor and Mao, 2019). However, we assume that particularly women could especially benefit from multimodal approaches, e.g., a day clinic, as they are confronted with special challenges due to their different roles: family, work and society. Moreover, these results indicate the importance of psycho-oncological interventions that focus on individual resources, behavioral change and support patients to take care of themselves and their limits (e.g., with mindfulness-based strategies). An expansion of supportive integrative medical services could thus have a positive impact both on individuals themselves and on their environment.

### 4.5. There is need for programs tailored to the needs of different age groups

This study demonstrated that more than half of the patients were affected by sleep-through disturbance. This matches results recently published by Voiss et al. for cancer survivors with up to 60% suffering from sleep problems and studies focusing on patients under treatment which found insomnia symptoms in up to 59% of cancer patients (Palesh et al., 2010; Savard et al., 2011; Voiss et al., 2019). In our study, the group aged 40–65 was most frequently affected by sleep disorders. Strikingly this age group also reported feeling stressed and dissatisfied with their own situation much more often. We also found an association between sleep disturbances and perceived personal energy level. It is comprehensible that poor nighttime sleep could be related to reduce wellbeing during the day, from increased feelings of stress up to increased susceptibility to depression. Several studies (Savard et al., 2011; Irwin et al., 2013; Lee et al., 2022) have already demonstrated this.

On the other hand, this age group reported the greatest motivation to learn a relaxation technique in the next 6 months and most frequently used self-care strategies and natural remedies for symptom relief. We hypothesize that the group of 40–65-year-olds might be affected in a special way due to, e.g., financial worries, future career and family responsibilities. Being confronted with a life-threatening disease in the most productive years of their lives could be a strong motivation to leaving no stone unturned to work toward the most positive further course possible. To our knowledge, there are some well-evaluated programs that include this age group (Faller et al., 2013), but none that are tailored to address the specific problems of this highly motivated age group in a personalized way.

### 4.6. Limitations of the study

The results must nevertheless be interpreted carefully. First, due to restrictions during the pandemic, we did not succeed in recruiting a representative patient population with respect to the frequencies of the entities as originally planned. Therefore, the distribution of disease sites of the participants is not representative of cancer incidence across sites. Thus, the findings are only representative of the groups sample and generalizability is limited.

Second, we used a custom-developed questionnaire based on a German Mind–Body Medicine Day Care Clinic program. This questionnaire was not validated. Third, the study question was very ambitious and this was at the same time a limitation of the study, as parameters relevant for patients were mixed with parameters relevant for the provider. However, the study approach was deliberately chosen to be very open and multifaceted, as this study may be understood from a clinical perspective as a screening for not yet sufficiently addressed needs of patients to become aware and thus has an exploratory character. For this reason, no hypotheses were formed, so that the subgroup analyses, for which a case number calculation based on hypotheses would actually be desirable, must be interpreted with caution and the results from this should only serve as an initial orientation for follow-up studies.

Agreement on pessimistic or optimistic thoughts does provide a mood picture. However, we cannot exclude the possibility that some patients were perhaps suffering, e.g., from an undiagnosed anxiety disorder or depression. Therefore, the statements used can only be understood as screening, and patients with a more pessimistic response behavior should be tested for psychological comorbidities using standardized instruments. Furthermore, the statements represent snapshots and, depending on the current individual situation, the results may also fluctuate in the short term so that longitudinal assessments should be more reliable for follow-up studies and in routine practice. Follow-up studies should optimally include more patients aged <39 years. Furthermore, a better differentiation must be made between patients with chronic course and patients with limited survival in the large group of palliative patients since these two groups are likely to differ significantly in symptom burden and needs.

Some patients abandoned the questionnaire, explaining it was too time-consuming. The effort required to complete the questionnaire may have led to more open-minded patients agreeing to participate. No data was collected on this. Furthermore, the impact of patients answering questions according to the principle of social desirability remains unclear.

### 4.7. Conclusion

To conclude, interventions and programs focusing on Mind–Body techniques, patient’s individual resources, with tools to explore impairing patterns of self-perception and cognitive biases, can be a valuable resource for oncology patients and should therefore be part of an integrative medical treatment concept. Especially for women and patients aged between 40 and 65 appropriately tailored programs would be reasonable regarding their special stress situation. Due to the demonstrated relations between a patient’s thoughts, feelings, and attitudes on the one hand and motivation for behavior change on the other hand, patients can also benefit from the sustainable implementation of a healthy lifestyle.

## Data availability statement

The original contributions presented in the study are included in the article/ Supplementary material, further inquiries can be directed to the corresponding author.

## Ethics statement

The studies involving human participants were reviewed and approved by the Ethics Committee of the Julius-Maximilian-University Wuerzburg on 15 April 2019 (study number 12/19-me). The patients/participants provided their written informed consent to participate in this study.

## Author contributions

CL and HE contributed to the study concept. JL, MW, LS, and AK contributed to the methodology. JL and CL were responsible for the formal analysis, drafted the manuscript, and carried out the investigation. MW, AK, LS, AQ, and HE critically reviewed and edited the manuscript. JL contributed to the data curation and visualization of the data. HE contributed to the supervision. All authors have read and agreed to the published version of the manuscript.

## Funding

This publication was supported by the Open Access Publication Fund of the University of Wuerzburg.

## Conflict of interest

CL: lecture fees from Celgene GmbH, Roche GmbH, Novartis Pharma GmbH, BMS GmbH & Co. KGaA, Mundipharma GmbH Co. KG, Merck KGaA.

The remaining authors declare that the research was conducted in the absence of any commercial or financial relationships that could be construed as a potential conflict of interest.

## Publisher’s note

All claims expressed in this article are solely those of the authors and do not necessarily represent those of their affiliated organizations, or those of the publisher, the editors and the reviewers. Any product that may be evaluated in this article, or claim that may be made by its manufacturer, is not guaranteed or endorsed by the publisher.
