# Supplementary material for: Mind–body-medicine in oncology—from patient needs to tailored programs and interventions: a cross-sectional study
Source: Front Psychol. 2023 Jul 6;14:1140693. doi: 10.3389/fpsyg.2023.1140693 (PMC10357839; doi:10.3389/fpsyg.2023.1140693)
Supplement: Supplementary file 1 [file Data_Sheet_1.DOCX]

Supplementary Material

Mind-Body-Medicine (MBM) in oncology - from patient needs to tailored programs and interventions – a cross-sectional study.

Jonas Leonhardt^1^, Marcela Winkler^2^, Anne Kollikowski^3^, Lisa Schiffmann^3^, Anne Quenzer^4^, Hermann Einsele^1^ and Claudia Löffler^1,3*^

*** Correspondence:**

Claudia Löffler: [Loeffler_C@ukw.de](mailto:Loeffler_C@ukw.de)

# Supplemental Tables

## Supplemental Table 1): Entities of interviewed Patients

| **Hematology** | n= 130 | **Gastroenterology** | n= 82 | **Gynecology** | n= 49 | **Dermatology** | n= 24 | **unclassified** | n= 9 |
| --- | --- | --- | --- | --- | --- | --- | --- | --- | --- |
| multiple myeloma | 78 (60%) | colorectal cancer | 29 (35%) | breast cancer | 34 (70%) | melanoma | 22 (92%) | sarcoma | 5 (56%) |
| other lymphatic cancer | 37 (29%) | pancreatic cancer | 22 (27%) | ovarian cancer | 6 (12%) | squamous cell carcinoma | 2 (8%) | others | 4 (44%) |
| leukemias | 8 (6%) | AEG and gastric cancer | 17 (21%) | tubal carcinoma | 3 (6%) |  |  |  |  |
| other | 7 (5%) | other | 14 (17%) | other | 6 (12%) |  |  |  |  |

**1.2.** **Supplemental Table 2): Questionnaire and Information about Missing Data**

(Rounding differences are not compensated when totals are calculated)

| Question | Answer | % | Total Answers  (max = 294) |
| --- | --- | --- | --- |
| **personal data** | | | |
| age | 25- 39y | 3 | 294 |
|  | 40- 65y | 48 |  |
|  | >65y | 49 |  |
| occupation | not retired | 56 | 285 |
|  | retired | 45 |  |
| **physical and mental well-being** | | | |
| What is your daily rhythm (when do you get up, when do you go to bed)? | mean time in bed= 8,8h /day | | 224 |
| Do you sleep well and restfully? | agree | 68 | 271 |
|  | disagree | 33 |  |
| Is it difficult to fall asleep? | agree | 29 | 272 |
|  | disagree | 71 |  |
| Is there a sleep-through disorder? | agree | 61 | 269 |
|  | disagree | 39 |  |
| Do you have to take medication regularly? If yes, which ones? | more than five different substances | 49 | 294 |
|  | less than five different substances | 51 |  |
| On a scale of 0-10 (0= I am very exhausted, 10 = I am feeling very strong) - on what energy level are you on average? | low energy level (< 4) | 14 | 267 |
|  | medium energy level (≥ 4 and < 7) | 48 |  |
|  | high energy level (≥ 7) | 38 |  |
| **Breathing** | | | |
| “I am aware that my breathing is related to my inner tension.” | agree | 72 | 279 |
|  | disagree | 28 |  |
| “I consciously pay attention to my breathing at least once a day.” | agree | 54 | 278 |
|  | disagree | 46 |  |
| “If not, "I plan to start in the next 30 days." | agree | 28 | 189 |
|  | disagree | 72 |  |
| **Nutrition** | | | |
| Do you eat a healthy diet for the most part, especially in view of your condition? | No and I don't plan to start in the next 6 months. | 71 | 276 |
|  | No, but I would like to start in the next 6 months. | 12 |  |
|  | No, but I plan to start in the next 30 days. | 4 |  |
|  | Yes, I have been eating healthily for less than 6 months. | 5 |  |
|  | Yes, I have been eating healthily for more than 6 months. | 7 |  |
| “I am aware that I can influence my well-being/health with what I eat.” | agree | 95 | 292 |
|  | disagree | 6 |  |
| “I usually eat mindfully (no TV, no newspaper), take at least 20 min. for a meal, sit down at a table for it, chew each bite thoroughly and enjoy the food.” | agree | 79 | 290 |
|  | disagree | 21 |  |
| “Stimulants are not food, but they can still be good for us if we enjoy them in moderation (e.g. 1-2 cups of coffee, 1 bar of dark chocolate, 0.1 liters red wine with a meal). I indulge in luxury foods:” | Never. | 8 | 284 |
|  | Several times a week. | 36 |  |
|  | Daily, but in moderation. | 53 |  |
|  | Daily, in larger quantities, sometimes instead of a meal. | 3 |  |
| “To cover my fluid needs, I drink at least 2 liters of water, herbal teas, juice spritzers daily.” | yes | 72 | 286 |
|  | no | 28 |  |
| Do you smoke or use drugs? | yes | 9 | 282 |
|  | no | 91 |  |
| **Exercise** | | | |
| Do you currently exercise regularly, i.e. for 30 min each on at least 4 days per week (walking, hiking, cycling...)? | No and I don't plan to start in the next 6 months. | 7 | 270 |
|  | No, but I would like to start in the next 6 months. | 7 |  |
|  | No, but I plan to start in the next 30 days. | 7 |  |
|  | Yes, I have been moving regularly for less than 6 months. | 6 |  |
|  | Yes, I have been moving regularly for more than 6 months. | 72 |  |
| “I have firmly built exercise into my daily routine (e.g. stairs instead of elevator).” | agree | 88 | 283 |
|  | disagree | 12 |  |
| “I exercise regularly (workout).” | agree | 30 | 280 |
|  | disagree | 70 |  |
| “Due to my illness I am physically very limited in movement.” | agree | 52 | 282 |
|  | disagree | 48 |  |
| **Relaxation** | | | |
| Do you currently perform conscious relaxation exercises such as autogenic training, progressive muscle relaxation, fantasy journeys, meditations or others on a regular basis? | No and I don't plan to start in the next 6 months. | 46 | 276 |
|  | No, but I would like to start in the next 6 months. | 11 |  |
|  | No, but I plan to start in the next 30 days. | 6 |  |
|  | Yes, I have been relaxing regularly for less than 6 months. | 6 |  |
|  | Yes, I have been relaxing regularly for more than 6 months. | 13 |  |
|  | On demand. | 18 |  |
| “I often feel tense, burdened and stressed.” | agree | 42 | 280 |
|  | disagree | 58 |  |
| “I have the impression that the stress load (e.g. internal tension) has a negative impact on my health.” | agree | 56 | 275 |
|  | disagree | 44 |  |
| „I am convinced that I can influence my inner tension.” | agree | 84 | 279 |
|  | disagree | 17 |  |
| **Thoughts - Feelings – Attitudes** | | | |
| “I think much and often about my disease.” | agree | 45 | 275 |
|  | disagree | 55 |  |
| If you are still in therapy: “There are things in my life to be grateful for.” | agree | 95 | 271 |
|  | disagree | 5 |  |
| “Being sick led to some good things in my life as well.” | agree | 48 | 247 |
|  | disagree | 52 |  |
| The following thoughts are very familiar to me and come to my mind more. Pick those answers you would agree with often (multiple answers possible). | “I need to function perfectly to do well. “ | 29 | 294 |
|  | “Everything is always up to me.” | 9 |  |
|  | “Life is unfair.” | 16 |  |
|  | “Sadness and pain are part of life, but there are always good times to follow.” | 60 |  |
|  | “I am satisfied with my life.” | 57 |  |
| **social network and occupation** | | | |
| “I feel well-integrated with my family and/or friends.” | agree | 96 | 288 |
|  | disagree | 4 |  |
| “I have family/friends/girlfriends that I can rely on. We help each other when we are in need.” | agree | 98 | 281 |
|  | disagree | 3 |  |
| „I enjoy my work and/or domestic tasks and I enjoy doing them." | agree | 87 | 282 |
|  | disagree | 14 |  |
| **lifestyle change** | | | |
| I have already successfully changed behaviors in my lifestyle (e.g. diet, exercise, relaxation) and have been able to maintain this for a long period of time. | agree | 66 | 282 |
|  | disagree | 34 |  |
| When I've changed a behavior in the lifestyle areas mentioned (e.g. diet, exercise, relaxation), I've often - after only a short time - ended up back in old habits. | agree | 37 | 256 |
|  | disagree | 63 |  |
| **national remedies** | | | |
| Have you already integrated a method from the spectrum of naturopathic "home remedies", such as water applications, medicinal herb or plant applications, regular sauna sessions or massages to strengthen your health into your everyday life? | No and I don't plan to start in the next 6 months. | 48 | 273 |
|  | No, but I would like to start in the next 6 months. | 15 |  |
|  | No, but I plan to start in the next 30 days. | 8 |  |
|  | Yes, I have been using natural remedies regularly for less than 6 months. | 5 |  |
|  | Yes, I have been using naturopathic home remedies regularly for more than 6 months. | 24 |  |
